# Development and validation of prognostic nomogram for elderly patients with clear cell renal cell carcinoma based on the SEER database

**DOI:** 10.1097/MD.0000000000035694

**Published:** 2023-10-20

**Authors:** Mingxin Lin, Cong Wang, Jianan Zhou

**Affiliations:** a The First Affiliated Hospital of Dalian Medical University, Dalian City, China.

**Keywords:** elderly clear cell renal cell carcinoma, nomogram, overall survival, SEER

## Abstract

This study sought to establish nomogram models of overall survival (OS) in patients with elderly clear cell renal cell carcinoma (ECCRCC). The Surveillance, Epidemiology, and End Results database provided data of the ECCRCC-afflicted patients diagnosed during the period from 2010 to 2015. This data was subsequently segregated into the training and validation sets randomly in a 7:3 ratio. The calibration curves, the receiver operating characteristic curves, the decision curve analysis and the Concordance index (C-index) were applied for the model evaluation. 9201 eligible cases from 2010 to 2015 were extracted; 6441 were included in the training cohort and 2760 in the validation cohort. The C-index for the training and validation sets were 0.710 and 0.709, respectively. The receiver operating characteristic and decision curve analysis curves demonstrated that nomograms outperformed the AJCC stage in predictive performance. Moreover, the nomogram was found to match closely with the actual observation, as indicated by the calibration plots. To make predictions with regard to the survival of the ECCRCC-afflicted individuals, and as a guide for treatment, the new nomogram could be used.

## 1. Introduction

One of the types of the most prevalent malignant tumors is the renal cancer. In the year 2021 in the United States itself, there were 13,780 new deaths with 76,080 new renal cancer cases.^[1]^ Approximately 70% of renal cell carcinomas are the most prevalent subtype of clear cell carcinoma.^[2]^ Compared to the other forms of renal cell cancer, the lowest survival rate is displayed by the Clear Cell Renal Cell Carcinoma (CCRCC).^[3]^ Patients afflicted below the age of 40 years ranged between 3% to 7%, while the median age is 65 years at the diagnosis of the renal cancer.^[4]^ Increasing age is the leading risk factor for renal tumors. It is commonly believed that elderly patients have poor prognoses.^[5]^ Despite the intensification of global population aging and the enhancement of medical care, the incidence of renal cancer in the elderly continues to rise.^[6]^ In order to guide clinical practice, it is necessary, based on the epidemiological characteristics of kidney cancer, to accurately assess the prognosis of the disease in elderly patients.

For assessing the prognosis of tumor patients, the frequent use of the tumor node metastasis (TNM) staging system of the United States Joint Commission has been done, subsequent to surgery.^[7]^ Nevertheless, the renal cell carcinoma prognosis was found as being influenced by various factors like treatment, size, sex, and age.^[8,9]^ The accuracy of patient survival and prognosis may be diminished by simple TNM stage. Therefore, the prediction model is gaining popularity due to its modeling simplicity and ability to incorporate multiple variables. Some academics have developed prediction models related to the survival and prognosis of renal cell carcinoma (RCC), enabling a good predictive value for the entire population and young adults.^[10,11]^ Additionally, some academics constructed the prediction of distant RCC metastasis.^[12,13]^ However, for the prediction of the survival outcomes in case of elderly clear cell renal cell carcinoma (ECCRCC), very few nomograms were used. For the prognosis of patients with elderly clear-cell renal cell carcinoma, a novel nomogram was developed and validated, basing on the extensive Surveillance, Epidemiology, and End Results (SEER) population database.

## 2. Methods

### 2.1. Patient selection

**Figure 1.**
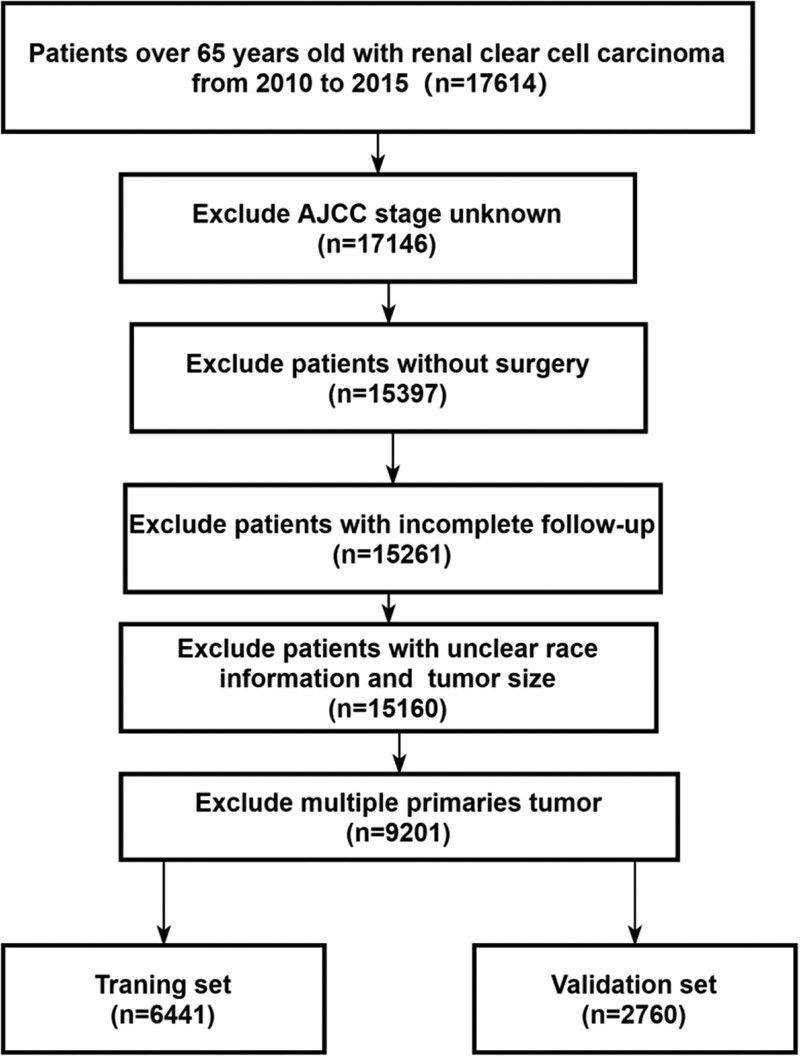
Flow diagram of ECCRCC patients with training and validation sets. ECCRCC = elderly clear cell renal cell carcinoma.

The clinical data of ECCRCC patients were downloaded from the SEER database using the SEER*Stat program. Between 2010 and 2015, 9201 ECCRCC patients were diagnosed and enrolled. The inclusion criteria were as follows: age ≥ 65 years old; histological type was confined to 8310/3 according to ICD-0-3; complete information on the American Joint Committee on Cancer (AJCC) stage; only one primary tumor case selected; surgery performed in each case; complete survival data; and compete clear information in race and tumor size. Random assignment to the validation and training cohorts was done of the eligible patients (7:3) (Fig. 1).

### 2.2. Clinical variables extracted for analysis

Age, gender, race, size, grade, 7th edition of AJCC stage, distant metastasis (bone, brain, liver, and lung), chemotherapy, and radiotherapy were clinical variables. The end of follow-up or death from all causes from the date of diagnosis, was defined as the period of overall survival (OS).

### 2.3. Statistical analyses

The chi-square test compares the clinical characteristics of the development cohort and validation cohort data. Univariate and multivariate Cox regressions were used to identify distinct variables, which were then output to RStudio software (version 4.1.2) to construct a nomogram model. With the application of the internal validation, calibration curves, decision curve analysis (DCA) curves, receiver operating characteristic (ROC) curves, and the concordance index (C-index), the evaluation of the predictive performance of the nomogram was done. Moreover, for the comparison of the nomogram model against the 7th TNM staging system of the AJCC in the validation and the development cohorts, the DCA and ROC curves were applied. Applying the maximum Jordan index and dividing the mortality rates into low and high-risk groups, the optimal cutoff value of the Kaplan–Meier survival curve was established. The differences between low and high-risk groups were determined using the log-rank test. Statistical significance was accorded to *P* values below .05.

## 3. Results

### 3.1. Baseline characteristics of ECCRCC patients

With 2760 patients being assigned to the validation cohort and 6441 to the training cohort, randomly, a total of 9201 patients were included finally in this study following the inclusion criteria (Fig. 1). While 7966 (86.6%) of all the patients were white, 5383 (58.5%) were male. Most were between 65 and 74 years old (6334, 68.8%). Most of the tumors were grade II. Bone metastasis rate was 2.1%, brain metastasis rate was 0.5%, liver metastasis rate was 0.7%, and lung metastasis rate was 4.1%. For tumor size, 22% were ≤ 29 mm, 42.8% were 30 to 59 mm, and 35.2% ≥ 60 mm. In case of any of the included variables, no differences were found to exist significantly between the validation and training cohorts (Table 1).

**Table 1:** Baseline demographic and clinical characteristics.

Variables	Total	Training cohort	Validation cohort	*P*
Total	(N = 9201)	(N = 6441)	(N = 2760)	
Age				
65–74	6334 (68.8)	4447 (69.0)	1887 (68.4)	.8080
75–84	2565 (27.9)	1785 (27.7)	780 (28.3)	
>85	302 (3.3)	209 (3.2)	93 (3.4)	
Gender				
Female	3818 (41.5)	2700 (41.9)	1118 (40.5)	.2163
Male	5383 (58.5)	3741 (58.1)	1642 (59.5)	
Race				
White	7966 (86.6)	5576 (86.6)	2390 (86.6)	.9680
Black	504 (5.5)	351 (5.4)	153 (5.5)	
Other	731 (7.9)	514 (8.0)	217 (7.9)	
Size				
0–29	2020 (22.0)	1432 (22.2)	588 (21.3)	.1797
30–59	3942 (42.8)	2780 (43.2)	1162 (42.1)	
60+	3239 (35.2)	2229 (34.6)	1010 (36.6)	
Grade				
Grade I	818 (8.9)	595 (9.2)	223 (8.1)	.2663
Grade II	4194 (45.6)	2915 (45.3)	1279 (46.3)	
Grade III	2500 (27.2)	1768 (27.4)	732 (26.5)	
Grade IV	638 (6.9)	436 (6.8)	202 (7.3)	
Unknown	1051 (11.4)	727 (11.3)	324 (11.7)	
Stage				
I	5720 (62.2)	4045 (62.8)	1675 (60.7)	.1207
II	754 (8.2)	504 (7.8)	250 (9.1)	
III	2031 (22.1)	1403 (21.8)	628 (22.8)	
IV	696 (7.6)	489 (7.6)	207 (7.5)	
Bone				
No	8982 (97.6)	6289 (97.6)	2693 (97.6)	.3887
Yes	190 (2.1)	135 (2.1)	55 (2.0)	
Unknown	29 (0.3)	17 (0.3)	12 (0.4)	
Brain				
No	9129 (99.2)	6395 (99.3)	2734 (99.1)	.4696
Yes	44 (0.5)	29 (0.5)	15 (0.5)	
Unknown	28 (0.3)	17 (0.3)	11 (0.4)	
Liver				
No	9113 (99.0)	6385 (99.1)	2728 (98.8)	.4224
Yes	61 (0.7)	39 (0.6)	22 (0.8)	
Unknown	27 (0.3)	17 (0.3)	10 (0.4)	
Lung				
No	8788 (95.5)	6164 (95.7)	2624 (95.1)	.3541
Yes	375 (4.1)	253 (3.9)	122 (4.4)	
Unknown	38 (0.4)	24 (0.4)	14 (0.5)	
Chemotherapy				
No/unknown	8721 (94.8)	6114 (94.9)	2607 (94.5)	.3836
Yes	480 (5.2)	327 (5.1)	153 (5.55)	
Radiation				
No/unknown	9022 (98.1)	6310 (98.0)	2712 (98.3)	.3922
Yes	179 (1.9)	131 (2.0)	48 (1.7)	

### 3.2. Nomogram construction

**Figure 2.**
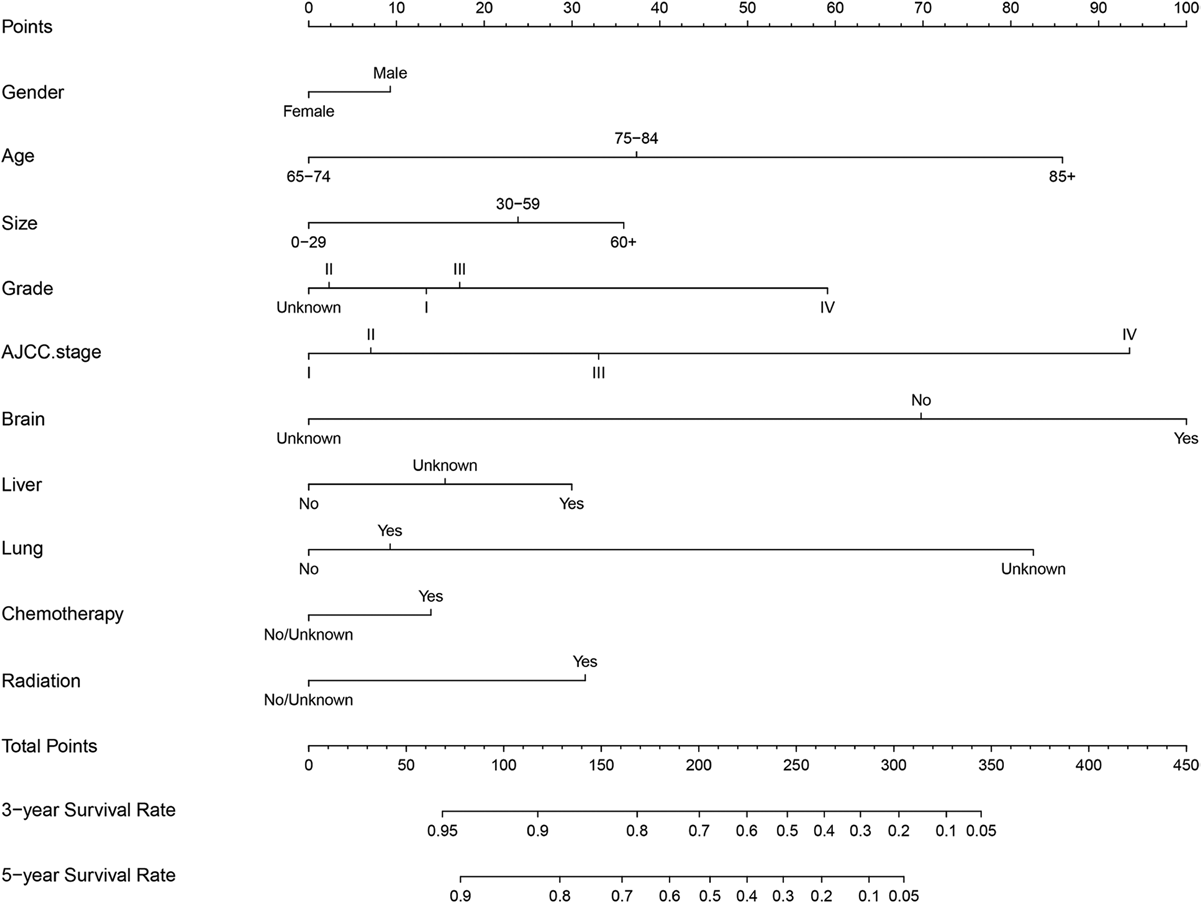
A nomogram predicting 3- and 5-year OS of ECCRCC patients in the training cohort. ECCRCC = elderly clear cell renal cell carcinoma, OS = overall survival.

Univariate Cox analysis identified gender, age, race, size, grade, AJCC stage, bone metastasis, brain metastasis, lung metastasis, liver metastasis, chemotherapy, and radiotherapy as significant predictors of OS rates in the training cohort. But race and bone metastasis were excluded in multivariate Cox analysis. (Table 2). Then, these ten independent variables were utilized to construct the 3- and 5-year OS nomograms (Fig. 2).

**Table 2:** Univariate and multivariate analysis of OS in the training cohort.

Variables	Univariate analysis		Multivariate analysis	
	HR (95% CI)	*P* value	HR (95% CI)	*P* value
Gender				
Female	Reference	–	Reference	–
Male	1.26(1.15–1.37)	<.001	1.15(1.05–1.26)	.002
Age				
65–74	Reference	–	Reference	–
75–84	1.65(1.51–1.81)	<.001	1.74(1.59–1.91)	<.001
85+	2.96(2.47–3.54)	<.001	3.55(2.69–4.26)	<.001
Race				
White	Reference	–	Reference	–
Black	0.91(0.75–1.11)	.348	1.11(0.92–1.35)	.283
Other	0.9(0.76–1.06)	.203	0.9(0.76–1.06)	.203
Size				
0–29	Reference	–	Reference	–
30–59	1.71(1.49–1.97)	<.001	1.42(1.23–1.64)	<.001
60+	3.32(2.89–3.8)	<.001	1.7(1.44–2.01)	<.001
Grade				
Grade I	Reference	–	Reference	–
Grade II	1.01(0.85–1.2)	.902	0.85(0.71–1.01)	.063
Grade III	1.73(1.45–2.06)	<.001	1.05(0.88–1.26)	.581
Grade IV	4.18(3.44–5.09)	<.001	1.97(1.6–2.43)	<.001
Unknown	1.16(0.94–1.42)	.168	0.82(0.66–1.01)	.060
AJCC stage				
I	Reference	–	Reference	–
II	1.53(1.29–1.8)	<.001	1.11(0.91–1.34)	.296
III	2.28(2.06–2.53)	<.001	1.63(1.44–1.85)	<.001
IV	7.73(6.85–8.72)	<.001	3.82(3.07–4.76)	<.001
Bone metastasis				
No	Reference	–	Reference	–
Yes	5.56(4.59–6.73)	<.001	1.15(0.87–1.53)	.33
Unknown	2.73(1.51–4.95)	.001	3.66(0.69–19.51)	.129
Brain metastasis				
No	Reference	–	Reference	–
Yes	11.3(7.66–16.68)	<.001	1.66(1.06–2.16)	.026
Unknown	2.43(1.34–4.39)	.003	0.17(0.03–0.89)	.036
Liver metastasis				
No	Reference	–	Reference	–
Yes	7.21(5.08–10.24)	<.001	1.58(1.09–2.28)	.015
Unknown	2.86(1.62–5.04)	<.001	0.89(0.25–3.12)	.855
Lung metastasis				
No	Reference	–	Reference	–
Yes	5.9(5.1–6.83)	<.001	1.19(0.96–1.49)	.118
Unknown	3.35(2.08–5.4)	<.001	3.08(1.56–6.05)	.001
Chemotherapy				
No/Unknown	Reference	–	Reference	–
Yes	4.66(4.08–5.33)	<.001	1.21(1.01–1.45)	.039
Radiotherapy				
No/Unknown	Reference	–	Reference	–
Yes	6.06(5–7.35)	<.001	1.47(1.11–1.94)	.007

### 3.3. Nomogram validation

**Figure 3.**
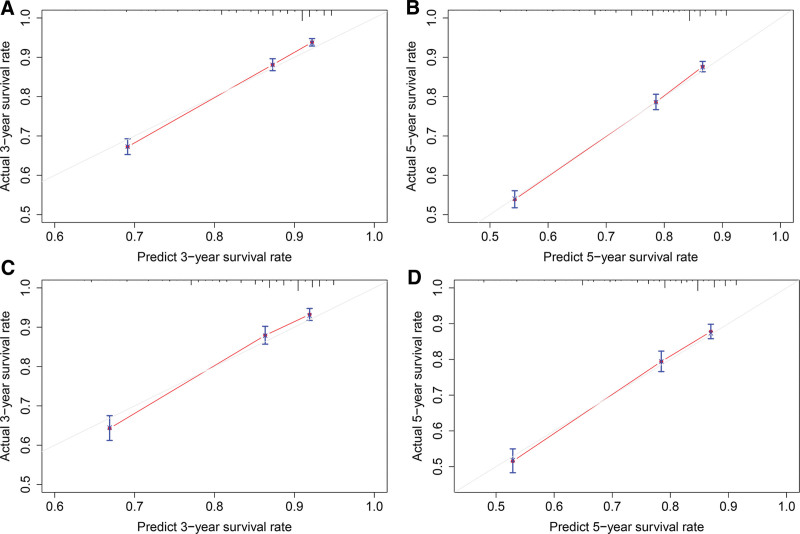
Calibration plots of OS nomogram model. (A, B) Calibration plots of 3-and 5-year OS in training set; (C, D) Calibration plots of 3-and 5-year OS in validation. OS = overall survival.

**Figure 4.**
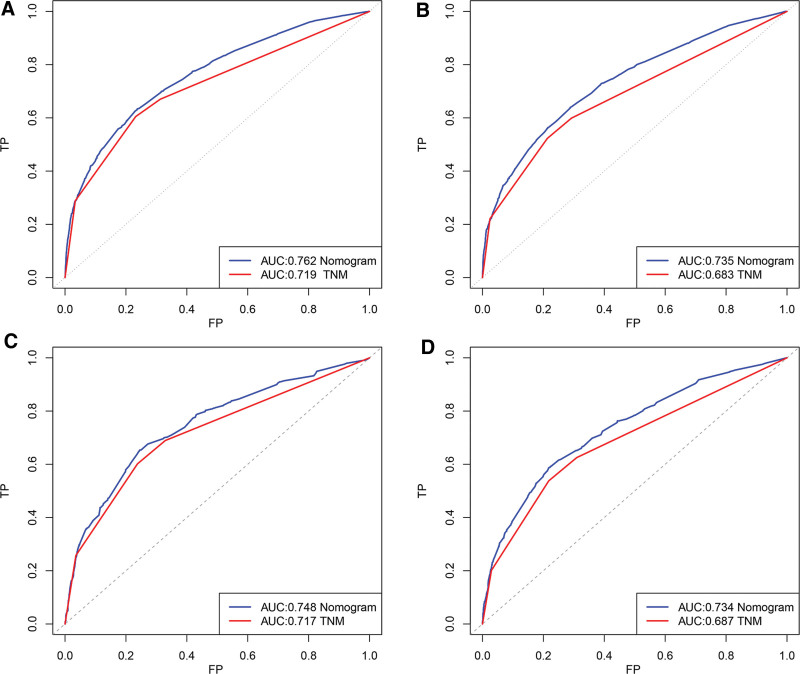
ROC curves for nomogram for predicting the 3- and 5-year OS of ECCRCC patients. (A, B) ROC curves in the training cohort. (C, D) ROC curves in the validation cohort. ECCRCC = elderly clear cell renal cell carcinoma, OS = overall survival, ROC = receiver operating characteristic.

**Figure 5.**
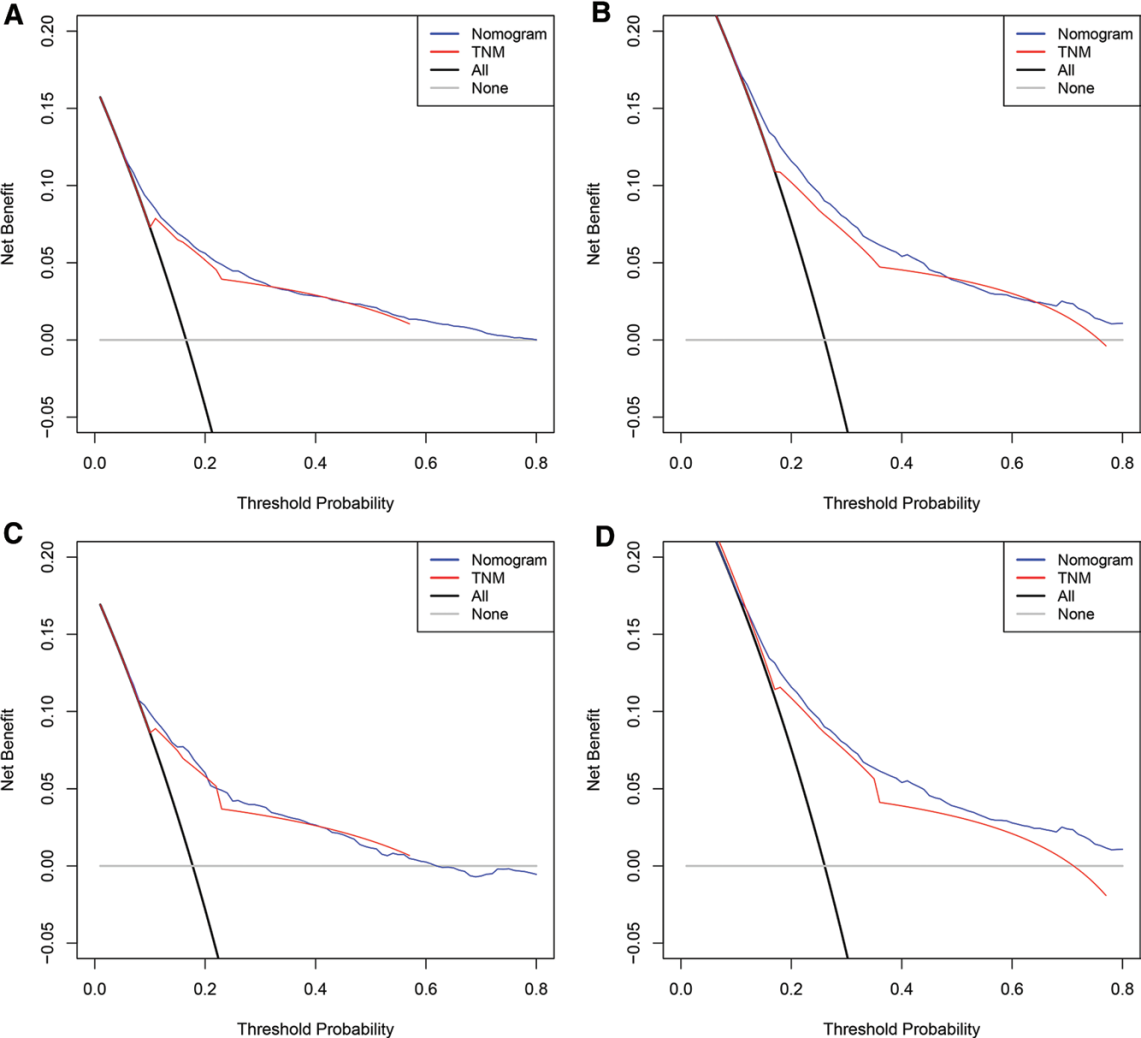
DCA of the nomogram for OS. (A, B) DCA of 3-and 5-year OS in training set;(C, D) DCA of 3-and 5-year OS in validation set. DCA = decision curve analysis, OS = overall survival.

Applying the calibration plots and the C-index, the external and internal evaluations of the nomogram model were done. In the training set, the C index of the OS nomogram was 0.710 (95% CI: 0.698–0.721), while in the validation set, it was 0.709 (95% CI: 0.691–0.726). Meanwhile, calibration charts demonstrated the model’s high reliability (Fig. 3). Comparing the nomograms with the traditional AJCC TNM stage method, the area under the ROC curve (AUC) was greater than the AJCC TNM stage method (Fig. 4). Greater net clinical benefits were predicted by the model as against those indicated by the TNM stage by the DCA results. Hence, a net positive benefit was indicated by the nomogram model results in predictions for the 3- and 5-year survivals of the elderly patients afflicted with ECCRCC (Fig. 5).

### 3.4. Survival analysis

**Figure 6.**
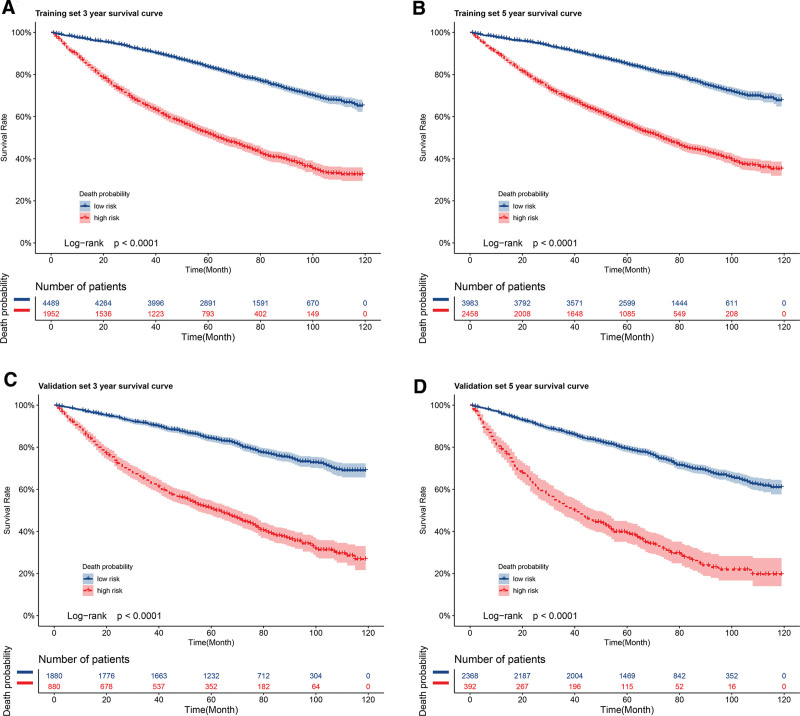
Predicted probability of overall survival by risk (A) 3-year mortality in training set (B) 5-year mortality in training set (C) 3-year mortality in validation (D) 5-year mortality in validation, shown using Kaplan–Meier curve.

Basing on the cutoff value, the prognostic nomogram model could define the groups of low and high-risk. In the training set, the optimal 3- and 5-year mortality cutoff points were 0.169 and 0.249. Figure 6A depicts the training set survival probability at three years for the low-risk (0.913; 95% CI: 0.91–0.92) and high-risk groups (0.654; 95% CI: 0.63–0.68). Figure 6B depicts the training set probability of survival at 5 years for the low-risk (0.85; 95% CI: 0.84–0.86) and high-risk groups (0.56; 95% CI: 0.54–0.58). In the validation group, the optimal 3- and 5-year mortality cutoff points were 0.167 and 0.278. In the validation cohort, the 3-year survival rate for low-risk patients was 0.91 (95% CI: 0.90–0.92), and for high-risk patients, it was 0.64 (95% CI: 0.61–0.67) (Fig. 6C). In the low-risk and high-risk groups, 5-year survival was 0.79 (95% CI: 0.78–0.81) and 0.39 (95% CI: 0.34–0.44), respectively (Fig. 6D). A statistically significant difference was observed between the low and high-risk groups among all the groups.

## 4. Discussion

There is widescale use of the nomogram presently in the prognosis of the oncology patients.^[14]^ With the use of a visual nomogram, the probabilities of survival of the elderly patients with CCRCC for 3 and 5 years could be predicted. Basing on the SEER database, this is the first study to develop and validate for the elderly kidney cancer patients, a model for clinical prognosis. Making the prediction effective clinically, the nomogram displayed convincing calibration plots with a higher C-index in this study. In addition, the AUC and DCA assessment systems yielded higher values than the AJCC TNM stages.

The incidence rate of renal cancer varies significantly by gender, with the male incidence rate being twice that of the female incidence rate.^[15]^ In our study, the incidence rate of male patients with CCRCC in the elderly is 1.4 times that of female patients, and male patients are found to have a poor prognosis. In vitro research confirmed that estrogen inhibits renal cell carcinoma cell progression through estrogen receptor-β activation.^[16]^ It may be the reason why women have a better prognosis. While the incidence of kidney cancer is higher in the black population than in the white population, the mortality rate is largely unaffected.^[8]^ Indicating no difference in survival outcomes significantly, more number of white patients were found to be afflicted with clear-cell renal cell carcinoma, according to the database of the SEER, in this research. Contributing to the enhanced prevalence in the elderly of the malignant diseases, aging was associated with the highly reproducible changes in the DNA methylation.^[17]^ Age was the third most significant factor in our nomogram, affecting the prognosis of patients. As is common knowledge, cancer is considered a disease of aging. The poor prognosis of renal cell carcinoma may be due to the decline of immune function and gene repair ability in the elderly.^[18]^ The size of the tumor remained a prognostic factor. As an anatomical factor, the size of the tumor increases the risk of local recurrence, according to the study.^[19]^ It affects the preservation of renal function after nephron-sparing surgery for kidney cancer, and a decline in renal function may contribute to an increase in all-cause mortality.^[20]^

In addition, 4 types of distant metastasis were incorporated into the nomogram of ECCRCC for SEER analysis. In all probability, almost 30% of patients already diagnosed with renal carcinoma stood the chance of being diagnosed with distant metastasis despite aggressive treatments for the primary tumor.^[21]^ Amounting to 27.6 and 43.6% of metastasis RCC in the bone and lung respectively, were observed to be the most common sites of metastasis of renal cancer. While the metastases were 4.4% in the incidence of liver and brain.^[22]^ We discovered that brain metastasis is the primary factor influencing ECCRCC prognosis. Wang et al developed a nomogram to aid physicians and patients in the active monitoring and follow-up of patients to prevent the spread of tumors to distant sites.^[13]^ AJCC stage had a significant impact on the prognosis of survival for ECCRCC patients. As demonstrated by a comparison between the prognostic model and the AJCC staging system, the AJCC stage plays a role in the survival prediction of ECCRCC patients. Our prediction model is based on enhancing clinical and demographic patient data according to the AJCC stage. According to Lau et al, the Fuhrman grade and tumor size are independently associated with metastasis-free and cancer-specific survival.^[23]^ Adding tumor grade as an additional prognostic variable to the prediction model can improve the prognosis model’s accuracy.^[24,25]^

The impact of chemotherapy and radiotherapy on the aspect of the prognosis of patients with ECCRCC being impacted by chemotherapy and radiotherapy, was also examined by the study. Patients having received chemotherapy and radiotherapy displayed poor prognosis as compared to those who had not, as per the results, while the RCC was observed to the most resistant tumor against radiotherapy and chemotherapy, both. Understandably, the most insensitive cancer types to radiotherapy were the renal cancers.^[26]^ Tian et al also discovered that radiotherapy and chemotherapy are ineffective against renal cancer in young patients.^[10]^ To arrive at a balance between the risks and benefits in the elderly, an evaluation of the physical condition of the patient was imperative, in the management of RCC, because of the prevalence of the basic diseases in them.^[6]^ Unfortunately, we did not extract hematology related indicators from SEER database to predict the prognosis of CCRCC. Bian et al^[27]^ developed nomogram via prothrombin time activity, prothrombin time, albumin/globulin ratio, platelets, sex, and fibrinogen in predicting recurrence-free survival time of renal cancer. Prothrombin time (%), prothrombin time (second),and fibrinogen could serve as independent risk factors for prediction of recurrence-free survival for CCRCC.^[27]^

Although our study talks about the nomogram with clinical information, however, the research on genes related to CCRCC has also been a hot topic in recent years. You et al demonstrated that elevated androgen receptor (AR) expression was positively correlated with tumor-originated vasculogenesis in CCRCC patients. The study revealed AR promoted vasculogenic mimicry formation in CCRCC cell lines via modulating lncRNA-TANAR/TWIST1 signals and targeting the axis could be suppress the CCRCC progression.^[28]^ The field of pyroptosis in kidney cancer has attracted increasing attention in recent years. The study found that different pyroptosis status CCRCC displayed distinct heterogeneity in multiple levels such as functional status, tumor microenvironment, alternation in genomics, response to chemotherapy and immunotherapy, and clinical outcomes.^[29]^ A significant correlation has been found between cell division cycle-associated genes and tumorigenesis and progression. Cell division cycle-associated genes (CDCAs) play vital roles for CCRCC. Especially CDCA5 and CDCA7 is already identified as an independent prognostic factors of the overall survival of CCRCC patients.^[30]^

Our investigation has several limitations. First, selection bias is unavoidable because this is a retrospective cohort study. Secondly, outside of the clinical data of the registry, there was no accessibility to the various significant prognostic factors like the humoral biomarkers, weight, and basic diseases. Finally, we conducted only internal data verification, hoping to obtain future external verification in the real world.

## 5. Conclusion

To conclude, to predict the survivals for 3 and 5 years in the ECCRCC patients, a nomogram was developed and validated. The new nomogram can be used as a simple clinical prediction tool to make individualized survival predictions for ECCRCC patients and has excellent clinical application value.

## Author contributions

**Conceptualization:** Mingxin Lin.

**Data curation:** Cong Wang.

**Investigation:** Mingxin Lin, Cong Wang.

**Methodology:** Cong Wang, Jianan Zhou.

**Software:** Cong Wang, Jianan Zhou.

**Supervision:** Jianan Zhou.

**Validation:** Cong Wang.

**Writing – original draft:** Cong Wang, Jianan Zhou.

**Writing – review & editing:** Mingxin Lin, Jianan Zhou.
